# The role of non-linear viscoelastic hydrogel mechanics in cell culture and transduction

**DOI:** 10.1016/j.mtbio.2025.102188

**Published:** 2025-08-09

**Authors:** Pascal Bertsch, Pasquale Sacco

**Affiliations:** a University of Fribourg, Department of Chemistry, Ch. du Musée 9, 1700, Fribourg, Switzerland; b University of Fribourg, Food Research and Innovation Center, Ch. du Musée 9, 1700, Fribourg, Switzerland; c University of Trieste, Department of Life Sciences, Via L. Giorgieri 5, Trieste, 34127, Italy

**Keywords:** Hydrogels, Rheology, Non-linear viscoelasticity, Mathematical modelling, Cell biology, Mechanotransduction

## Abstract

The mechanical complexity of the extracellular matrix (ECM) is central to how cells sense and respond to their environment, yet hydrogel design has often focused narrowly on stiffness. Emerging evidence highlights the importance of viscoelastic stress relaxation and plasticity in cell mechanotransduction. However, a key aspect remains underexplored: non-linear viscoelasticity, where stress relaxation and plasticity depend on the magnitude of applied stress or strain. In this perspective, we examine how such non-linear mechanical behaviors manifest in widely used hydrogels and discuss their biological relevance. We present experimental approaches, including oscillatory shear rheology, to detect non-linear viscoelastic effects, and introduce mathematical modeling approaches to interpret these behaviors. We find evidence in literature that several hydrogels commonly used in cell culture exhibit non-linear viscoelasticity occurring at stress and strain levels relevant to cell-generated forces. Specifically, both softening and stiffening hydrogels were found to exhibit accelerated stress relaxation or increased plasticity due to nonlinear viscoelasticity. By viewing non-linearity as a tunable design parameter, future hydrogel systems may better recapitulate the dynamic mechanical feedback loops cells experience in native tissues. This perspective encourages a paradigm shift in biomaterial design, integrating non-linear viscoelasticity into the next generation of ECM-mimetic hydrogels for cell culture and regenerative applications.

## Graphical abstract

**Figure:**
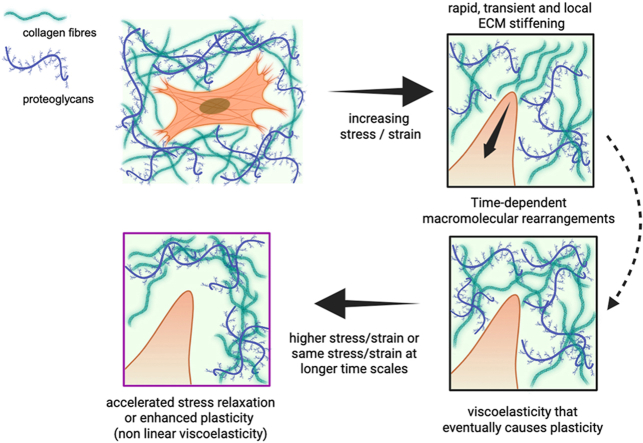

## 1 Introduction

Understanding how cells probe their surrounding microenvironment and convert biophysical (e.g., mechanical) into biochemical cues via mechanotransduction plays a fundamental role in the understanding of tissue development, homeostasis and regeneration [1]. Early studies on cellular mechanotransduction have primarily focused on linear elastic stiffness, the slope of the linear stress-strain response, of 2D substrates as a key material trait [[2], [3], [4]]. Standard glass or tissue culture plastic, albeit routinely used, are often not suitable for *in vitro* cell culture models to resemble the mechanics of living tissues as their high stiffness can lead to unnatural cell polarization, gene expression or uncontrolled differentiation. This has led to the emergence of biomimetic extracellular matrices (ECMs) in the form of hydrogels as versatile material platforms for cell culture and tissue engineering that can cover a wide range of stiffness [3,[5], [6], [7]]. Moreover, hydrogels have greatly facilitated the transition to 3D cell culture which better reflects the 3D microenvironment of the ECM in terms of mechanical properties, confinement and distribution of adhesion ligands [[6], [7], [8], [9], [10]]. The dynamic nature of hydrogels has also widened the perspective of mechanical cues beyond linear elastic stiffness, with hydrogels being designed to mimic the non-linear elasticity of living tissues, and more recently also time-dependent stress relaxation or permanent deformation, i.e., mechanical plasticity [[11], [12], [13], [14], [15], [16]]. Viscoelastic stress relaxation or plasticity of hydrogels have added a new dimension to the understanding of cellular mechanotransduction. Such viscoelastic effects are characteristics of living tissues and were already demonstrated to affect cell activity in terms of spreading [[17], [18], [19], [20]], migration [15,21,22], proliferation [17,18], and differentiation [17] and play a role in tissue regeneration [23], organoid culture [24,25], and disease [26,27]. A possible explanation is that viscoelasticity is a crucial property of living cells which themselves exhibit frequency dependent storage and loss moduli and non-linear mechanics, including stiffening and softening behavior [28]. This intrinsic property is attributed to biological structures such as the cytoskeleton, the cortex, the cytoplasm and the cell nucleus. Considering that cells interact dynamically with their surrounding microenvironment through a reciprocal mechanical feedback, nonlinear viscoelastic effects are expected both intra- and extracellularly. We anticipate that hydrogels entering the non-linear regime can have a pronounced effect on their viscoelastic stress relaxation or plasticity. Hence, we here discuss the role of viscoelasticity in the non-linear regime, an effect that has rarely been considered in literature but could offer new opportunities for the design of responsive hydrogels for cell and organoid culture.

## 2 Non-linear viscoelastic hydrogels in cell culture

### 2.1 Overview of non-linear viscoelasticity and relevant techniques

**Fig. 1:**
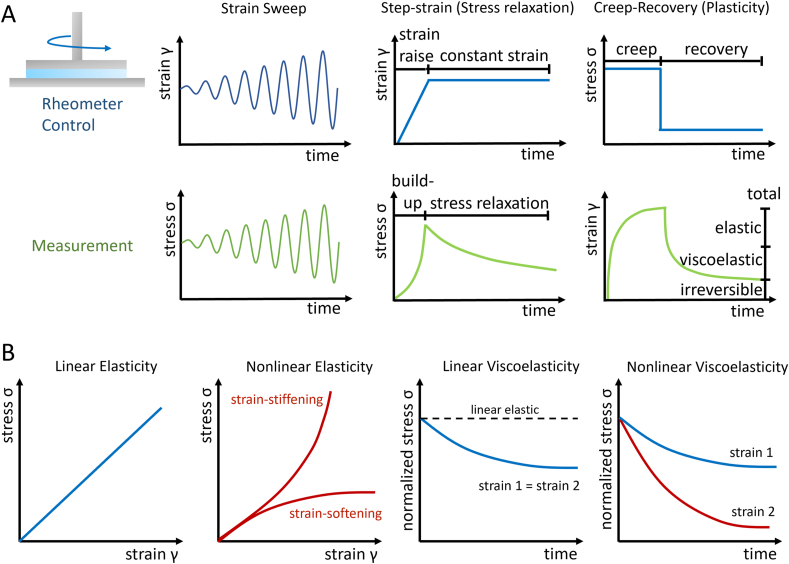
A) rheometer control and measured parameters for strain sweeps to determine stress-strain response, step-strains to determine viscoelastic stress relaxation, and creep-recovery experiments to determine viscoplasticity of materials. B) Schematic overview of linear and non-linear elasticity as well as linear viscoelasticity showing the same stress relaxation at two different strains or the same strain at two different points in time and non-linear viscoelasticity showing strain-dependent stress relaxation.

The mechanical testing of hydrogels involves the application of an external deformation and the determination of the resulting stress, or vice-versa. While uniaxial compression has long been the gold standard for determining the stiffness of hydrogels, rotational rheology is gaining importance for the study of viscoelastic effects as it can operate in a wide range of strain and frequency. A brief discussion on different deformation modes and length scales is provided below. Fig. 1A provides an overview of relevant rheological protocols for non-linear viscoelastic hydrogels. The stress-strain response of hydrogels is commonly determined by strain-controlled strain sweeps, where the oscillatory strain is gradually increased using a constant frequency and the required stress determined by the rheometer. Stress-controlled stress sweeps can be beneficial for certain applications, e.g., for determination of the yield stress of injectable or printable hydrogels [29,30]. After identifying the linear stress-strain range, the loss tangent tanδ derived from standard frequency sweep tests is a useful parameter to derive information about linear viscoelasticity, which is in the order of 10–20% at 1 Hz for living tissues [31]. Strain or stress sweeps are of particular interest considering that cells progressively access the non-linear stress-strain regime, sensing their surrounding microenvironment through cycles of polymerization/depolymerization of their cytoskeleton [14]. Time-dependent viscoelastic stress relaxation is determined in step-strain experiments, where the strain is rapidly increased and kept constant over time to determine the resulting stress build-up and subsequent relaxation. Mechanistically, stress relaxation in hydrogels derives from network rearrangements or polymer disentanglement and sliding and is most pronounced in hydrogels with non-covalent or dynamic covalent interactions [[32], [33], [34], [35], [36]]. An increasingly exploited strategy is the combination of covalent and non-covalent bonds (dual cross-linked hydrogels) or the incorporation of a second polymer (double network hydrogels) or freely movable particles [[37], [38], [39]]. Hydrogel plasticity is determined in creep-recovery experiments, where the hydrogel is exposed to a stress for a certain time followed by cessation of stress, measuring the resulting strain and its recovery, distinguishing the instantaneous elastic, transient viscoelastic, and irreversible plastic deformation. The degree of plasticity is defined as the ratio of irreversible strain and total strain (eq. (1)):

eq. 1 $$Degree\;of\;Plasticity=\frac{\gamma_{irreversible}}{\gamma_{total}}$$

Plasticity is a result of irreversible structural changes in the hydrogel matrix under load, such as bond breaking and reformation in covalent hydrogels or polymer stretching and pull-out in non-covalent hydrogels [[40], [41], [42]]. Hydrogels are typically linear elastic at low strain or very short time-scales, meaning that the applied strain and resulting stress are linearly proportional. However, with increasing strain, hydrogels may exhibit non-linear elasticity and show a ‘strain-softening or ‘strain-stiffening’ behavior (Fig. 1B). In the linear elastic regime, hydrogels also exhibit a linear viscoelastic behavior, and their viscoelastic response is the same for two different strains within the linear regime, or for the same strain measured at two different time points (time invariance) [43]. If one of the two conditions is not met, the hydrogels exhibit non-linear viscoelasticity and their stress relaxation or plasticity become a function of strain or time. We will henceforth primarily focus on non-linear viscoelasticity in the non-linear stress-strain regime.

### 2.2 Non-linear hydrogel mechanics can result in non-linear viscoelasticity

**Fig. 2:**
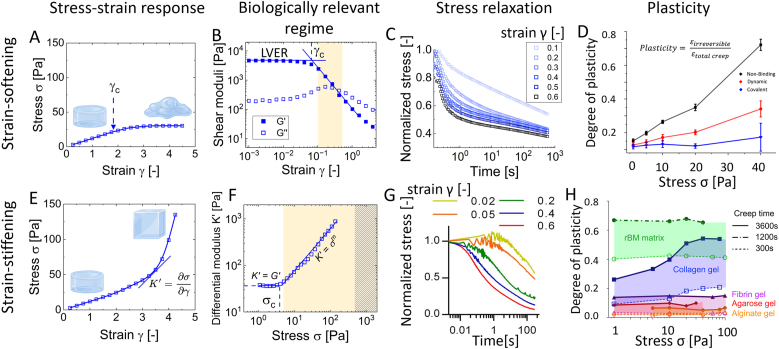
A) Typical stress-strain response of strain-softening hydrogels indicating critical strain γ_c_. The illustrations of hydrogels are “Created in Biorender. Sacco, P (2025) https://BioRender.com/w5tefc5 and https://BioRender.com/ncb153l ”. B). Strain sweep of strain-softening hydrogels depicting G′ and G″ as a function of strain indicating the linear viscoelastic regime (LVER) and γ_c_. The yellow regime indicates the strain range typically induced by cells. C) Normalized stress relaxation over time at increasing strain for strain-softening hydrogels depicted in B). Both replotted from Ref. [45] D) Plasticity of a set of alginate hydrogels designed to exhibit equivalent stress relaxation but altering plasticity, including non-linear viscoelastic hydrogels with increasing plasticity as a function of strain. Reprinted from Ref. [20], Copyright 2020 National Academy of Sciences. E) Typical stress-strain response of strain-stiffening hydrogels indicating the differential modulus K’. The illustration of hydrogels are “Created in Biorender. Sacco, P (2025) https://BioRender.com/tyd1a8j”. F). K′ as a function of stress for a strain-stiffening hydrogel with K’ = G′ in the LVER and power law increase K’ = σ^m^ beyond critical stress σ_c_. Replotted from Ref. [55]. The yellow regime indicates the stress range corresponding to traction stresses typically exerted by cells, while patterned areas correspond to peak stress observed at cell protrusions or during collective cell migration or mitosis. G) Normalized stress relaxation over time at increasing strain for strain-stiffening collagen hydrogels. Reprinted from Ref. [68], Copyright 2016 National Academy of Sciences. H) Plasticity as a function of creep stress and time for various hydrogels commonly used cell culture in cell culture with strain-stiffening collagen hydrogels exhibiting non-linear viscoelasticity. Reprinted from Ref. [16], (Copyright 2016), with permission from Elsevier. (For interpretation of the references to colour in this figure legend, the reader is referred to the Web version of this article.)

Fig. 2 provides an overview of non-linear hydrogel mechanics and its effect on viscoelastic stress relaxation and plasticity. Fig. 2A depicts the typical behavior of a strain-softening hydrogel that begins to deviate from a linear stress-strain response at the critical strain $\gamma_{c}$ above which the hydrogel ceases to respond elastically and begins to deform plastically. The mathematical modelling of strain-softening data and determination of $\gamma_{c}$ are further discussed below. Plotting the corresponding shear moduli as a function of strain (Fig. 2 B) allows the visualization of the linear viscoelastic regime (LVER) of hydrogels and experimental approximation of $\gamma_{c}$ (or critical stress $\sigma_{c}$ if performed in stress-controlled stress sweeps) at the intercept of the horizontal line through $G^{\prime }$ in the LVER and power-law fit of $G^{\prime }$ beyond $\gamma_{c}$ or $\sigma_{c}$ [30]. Viscoelastic effects in the LVER can be identified through standard frequency sweep tests by the loss tangent parameter, that is tanδ=G″G′, at a singular frequency, typically 1 Hz.

In the region of high strain amplitude beyond $\gamma_{c}$ or $\sigma_{c}$ the storage modulus $G^{\prime }$ decreases with increasing deformation and the interpretation of the results can be ambiguous. While many biomaterials exhibit evident strain-softening, their non-linear elastic response within a single oscillation cycle (described by a Lissajous curve), is often reported to exhibit strain-stiffening. This paradox between strain-softening and stiffening response has been clarified [44], suggesting that the materials do in fact soften, and the apparent stiffening is a consequence of the use of a tangent modulus that represents a local effect.

Entering the non-linear regime beyond $\gamma_{c}$ or $\sigma_{c}$ in strain or stress sweeps are a good predictor for non-linear viscoelastic stress relaxation [39,45]. Hydrogels depicted in Fig. 2B were designed to exhibit a strain-softening behavior at strains relevant for cells [45], which is in the range of γ = 10–50% as indicated in the graph [15,[46], [47], [48], [49], [50]]. This non-linear strain-softening was directly correlated with non-linear viscoelasticity and resulted in a considerably accelerated stress relaxation at increasing strain, as shown in Fig. 2C. Regarding plasticity, Grolman et al. [20] designed a set of alginate hydrogels that exhibited equivalent stress relaxation dynamics but varying degrees of plasticity by introducing poly(ethylene glycol) (PEG) spacers to unravel the effect of hydrogel plasticity on cell spreading. The plasticity of alginate hydrogels with dynamically bound or unbound PEG increased as a function of creep stress in contrast to covalently bound PEG (Fig. 2D). It was not specifically mentioned that respective hydrogels were stress-softening, although alginate hydrogels usually show a softening behavior at increasing stress or strain [[51], [52], [53]].

Fig. 2E depicts the typical behavior of a strain-stiffening hydrogel. The mathematical modelling of strain-stiffening hydrogels is further addressed below. Experimentally, strain-stiffening hydrogels can be described by the differential modulus $K^{\prime }$ which denotes the rate of stiffening, K′=∂σ∂γ. At low stress or strain in the LVER $K^{\prime }$ is equal to $G^{\prime }$. Beyond $\sigma_{c}$, $K^{\prime }$ is described by the stiffening index, m, according to $K^{\prime }={\;\sigma }^{m}$ (Fig. 2F) [54,55]. It is not straightforward to determine the stress regime relevant for cells. The traction stresses exerted by cells on their environment can be assessed by bead or matrix displacement and typically range from a few Pa to hundreds of Pa depending on cell and matrix type [46,49,50,56,57]. However, peak traction stresses can reach up to 1–5 kPa at lamellipodia or focal adhesions [46,58,59] or during collective cell migration [60] or mitosis [61]. The respective ranges are indicated in Fig. 2F. While it is already challenging to determine the strains and stresses exerted by cells, a key question in this regard is to what extent macroscopic rheology or indentation are representative of cellular length scales. There has recently been increased efforts to characterize soft tissues and hydrogels by micro- or nano-rheology which could bridge this gap [[62], [63], [64], [65], [66], [67]]. López-Serrano et al. [67] provided an interesting overview of hydrogel mechanical characterization at different length-scales and deformation modes, i.e., macroscopic compression, shear-rheology, and micro-rheology using atomic force microscopy, finding the same overall trends despite variations in absolute values. However, some material properties can vary significantly depending on deformation mode and length scale. For instance, viscoelastic hydrogels with the same shear modulus can exhibit considerably different compressive stiffness, indicating that material properties may be decoupled for viscoelastic hydrogels [39]. Hence, understanding how cellular length scales and deformations correlate with macroscopic testing, e.g., rheology or indentation, is one of the key bottlenecks in the understanding cell-material interactions.

Nam et al. investigated several hydrogels commonly used in cell culture in terms of stress relaxation [68] and plasticity [16], as visualized in Fig. 2G and H, respectively. Of the tested materials, strain-stiffening collagen and fibrin showed non-linear viscoelasticity and exhibited an accelerated stress relaxation at increased strain, as also reported elsewhere for collagen [39]. In terms of plasticity, only the plasticity of strain-stiffening collagen hydrogels was non-linear and increased as a function of creep stress (Fig. 2H). Other materials, i.e., fibrin, low-gelling temperature agarose, polyacrylamide, alginate, and reconstituted basement membrane, did not exhibit non-linear viscoelasticity in either stress relaxation or plasticity (note the difference to alginate hydrogels in presence of unbound PEG which show non-linear plasticity (Fig. 2D)). Non-linear viscoelasticity in the form of plasticity was, however, observed elsewhere for methylated high-gelling agarose hydrogels [13]. A non-linear increase in plasticity as a function of stress was also reported for polyisocyanide hydrogels [69].

In summary, Fig. 2 provides evidence that hydrogels may exhibit non-linear elasticity, and most importantly, non-linear viscoelasticity in strain or stress regimes relevant for cells. Such non-linear viscoelasticity, the possibility that hydrogel stress relaxation or plasticity can be a function of strain and/or stress, have largely been neglected as a design parameter for hydrogels in cell culture or tissue regeneration. Interestingly, based on the currently limited studies reporting non-linear viscoelasticity in hydrogels, we found that both strain-softening and strain-stiffening in hydrogels result in accelerated stress relaxation or increase in plasticity.

## 3 Mathematical modelling of non-linear hydrogel mechanics

Mathematical modeling helps to derive critical parameters to identify the onset of the non-linear region in strain-softening or -stiffening hydrogels. A typical oscillatory rheological experiment to derive such information are stress or strain sweep experiments, as shown in Fig. 1A, and respective hydrogel responses are depicted in Fig. 2A and E for strain-softening and strain-stiffening hydrogels, respectively.

### 3.1 The case of strain-softening behavior

Although “*there is no unique and rigorously motivated criterion allowing a yield stress to be determined from oscillatory data*” [70], three main strategies can be undertaken for calculating this critical strain/stress: (*i*) the point at which $G^{\prime }=G^{\prime \prime }$; (*ii*) by fitting the experimental points of $G^{\prime }$ using a power-law function above the critical stress and intersecting the latter with the linear response of the elastic modulus below the yield point (Fig. 2B); (*iii*) by the intersection of a line with unit slope at low strain with a power-law equation at large deformations using a typical stress-strain plot. We refer to the work of Bonn and co-workers for a more detailed description of the calculation of yield stress using different methods with conventional rheometers that have been used in the literature for a variety of materials [30]. Here we focus on the calculation of the yield point using a single equation in a typical stress-strain plot under the condition of using oscillatory rheology with a standard frequency of 1 Hz (or 6.28 rad/s) [13]. A typical stress-strain response of a strain-softening hydrogel is shown in Fig. 2A. Experimental points can be modeled, from the phenomenological point of view, using eq. (2):

eq. 2 $$\sigma =\frac{G}{1+b\gamma }\gamma$$

where σ represents the stress, γ is the strain while G and *b* are fitting parameters. G is the shear modulus at $\gamma_{\to 0}$ (eq. (3)):

eq. 3 $$G=lim_{\gamma \to 0}\frac{d\sigma }{d\gamma }$$

The critical strain, $\gamma_{c}$, which marks the onset of the non-linear softening (plastic) behavior, is defined according to eq. (4):

eq. 4 $${\frac{d\sigma }{d\gamma }|}_{\gamma =\gamma_{c}}=0.95\;lim_{\gamma \to 0}\frac{d\sigma }{d\gamma }$$

Therefore, eq. (4) can be rewritten as eq. (5):

eq. 5 $${G|}_{\gamma_{c}}=0.95\;G$$

Combining eq. (5) with eq. (2), $\gamma_{c}$ can be determined as eq. (6):

eq. 6 $$\frac{G}{1+b\gamma_{c}}=0.95\;G$$

Therefore, $\gamma_{c}$ is (eq. (7)):

eq. 7 $$\gamma_{c}=\frac{0.05}{0.95\;b}$$

Now one can assume arbitrarily a strain falling into non-linear softening region, $\gamma_{\max}$, and calculate the linear elastic energy loss, $E_{l}$, which is the difference between the theoretical elastic energy calculated for a linear stress-strain relationship and the experimental elastic energy from $\gamma_{c}$ to $\gamma_{\max}$ as (eq. (8))

eq. 8 $$E_{l}=\int_{\gamma_{c}}^{\gamma_{\max}}\left({\left(\frac{d\sigma }{d\gamma }\right)|}_{\gamma =0}-{\left(\frac{d\sigma }{d\gamma }\right)|}_{\gamma }\right)\gamma d\gamma \;with\;\gamma_{\max}\gg \gamma_{c}$$

$E_{l}$ reflects the plastic deformation of the hydrogel. Therefore, the higher $E_{l}$ the higher the network plasticity. Eq. (8) is therefore an alternative to eq. (1) for estimating the degree of plasticity of a hydrogel network under oscillatory shear stimulation.

### 3.2 The case of strain-stiffening behavior

A typical stress-strain response of a strain-stiffening hydrogel is shown in Fig. 2E. In addition to considering a typical plot of differential modulus, $K^{\prime }$, as a function of applied shear stress, as shown in Fig. 2F, where the change in curve slope gives an idea of the critical stress required to enter the non-linear stress-strain region, the use of deformation-dependent strain energy functions, as proposed by Erk and coworkers, represents a very useful and versatile model in accurately predicting the non-linear elasticity of biological networks of actin, collagen, fibrin, vimentin, neurofilaments, and that of hydrogel polymer networks [71]. Here, eq. (9) describes the strain energy density, *U*, as

eq. 9 $$U=\frac{G_{0}}{2}J^{*}\left[\exp\left(\frac{J_{1}}{J^{*}}\right)-1\right]\;;\;J_{1}=\lambda_{1}^{2}+\lambda_{2}^{2}+\lambda_{3}^{2}-3$$

where $G_{0}$ is the small-strain shear modulus and $\lambda_{1}$, $\lambda_{2}$ and $\lambda_{3}$ are the principal extension ratios. Considering that $J_{1}$ is closely related to the first strain invariant, $I_{1}$, according to eq. 10

eq. 10 $$J_{1}=I_{1}-3$$

the only fitting parameter in eq. (9) results, therefore, $J^{*}$, which can be regarded as the characteristic value of $J_{1}$, above which strain-stiffening becomes dominant. Assuming an incompressible material with

eq. 11 $$\lambda_{1}^{2}{\;\lambda }_{2}^{2}\;\lambda_{3}^{2}=1$$

which undergoes shear deformation in a typical 1–2 plane

eq. 12 $$\lambda_{3}=1\;;\;\lambda_{1}=\lambda_{2}^{-1}$$

eq. (9) can be rewritten in terms of shear strain, γ, as (eq. (13))

eq. 13 $$U_{shear}=\frac{G_{0}}{2}J^{*}\left[\exp\left(\frac{J_{1}}{J^{*}}\right)-1\right]\;;\;J_{1}={\left(\lambda_{1}-\lambda_{2}\right)}^{2}=\gamma^{2}$$

If one differentiates $U_{shear}$ with respect to γ, the following equation for the shear stress, τ, can be derived (eq. (14))

eq. 14 $$\tau =G_{0}\gamma \exp\left(\frac{\gamma^{2}}{J^{*}}\right)$$

The critical value of strain at which stiffening becomes dominant, $\gamma^{*}$, is calculated as (eq. (15))

eq. 15 $$\gamma^{*}=\sqrt{J^{*}}$$

## 4 Non-linear hydrogel mechanics in cell mechanotransduction

### 4.1 Overview of cell mechanotransduction and sensing

The first part of this section provides a brief overview of the main mechanotransduction mechanisms identified modulating the elasticity or viscoelasticity of 2D substrates or 3D microenvironments. Meticulous reviews discuss this topic in more detail [2,4,9,27,72]. Cells interact with their surrounding ECM through integrins or simple volume expansion, transmit forces and measure the resulting mechanical feedback. The end result is the intracellular transduction of biophysical cues associated with the ECM. Under 2D conditions, cells probe the underlying substrate stiffness through the myosin-actin-integrin axis. Adaptor proteins such as talin, tensin, filamin or actinin play a key role in connecting integrins to the actomyosin machinery [72]. After binding to ECM proteins cells begin to generate forces like an engine due to actomyosin contractility and transmit them to the outside. Depending on this mechanical feedback, intracellular mechanotransduction processes are elicited, resulting in the unfolding of talin and the subsequent binding of vinculin [73], the formation and growth of focal adhesions [74], the formation of stress fibers, the alteration of lamin A expression [75], the regulation of transport across nuclear pores [76], and the nuclear translocation of the Yes-associated protein (YAP) and the transcriptional coactivator with PDZ-binding motif (TAZ) [77], leading to the activation of different transcriptional pathways. As far as actin polymerization processes are concerned, such as spreading, filopodial extension or lamellipodial protrusions, there is a general consensus that cells gauge the stiffness of the underlying substrate, and this can be interpreted in terms of the ‘molecular clutch model’, where molecular clutches link actin to the substrate and mechanically resist myosin-driven actin retrograde flow [78]. The molecular clutch model can be eventually generalized in the case of viscoelastic substrates [79]. Interestingly, recent discoveries have shown that mechanosensing might not depend on the maturation of focal adhesions, but mainly on the distances between the anchoring points that the cells use to attach highly stiff polyacrylamide substrates (shear modulus around 1.3 MPa). Specifically, large nanospacing (>70 nm) between adhesive peptide sequences such as RGD promote the dissolution of integrin clusters, the increase of cytoplasmic actin dynamics and the translocation of globular actin into the nucleus with subsequent polymerization into filamentous structures. This increases nuclear tension, promotes the remodeling and accessibility of chromatin and causes the release of the YAP from the SWI/SNF complex, which leads to the activation of osteogenic genes [80]. To better mimic the dynamics of the extracellular microenvironment, the use of dynamic light-responsive hydrogels with controlled stiffening and softening cycles at variable frequencies rather than static substrates has helped to gain crucial insights into the dynamics of molecular clutch [81]. In particular, it was found that the chains of the molecular clutch disassemble when the substrate softens, causing signaling molecules such as pFAK to detach from the molecular clutch and migrate into the cytoplasm of the cell. When the substrate quickly becomes rigid again, the molecular clutch chains reassemble and the signaling molecules in the phosphorylated forms reattach to the molecular clutch chains. This leads to an increased presence of both pFAK and pMyosin IIa on the clutches, which ultimately leads to increased traction forces. Under 3D conditions, mechanotransduction of cells is mediated by integrins or ion channels that are activated after cell volume increase [9]. For integrin-mediated signaling pathways, a comparison of 2D and 3D systems reveals some analogies in terms of cell-ECM interactions. However, crucial differences are mainly observed in the polymerization rate of actin or the formation of focal adhesions. Moreover, there is little information on downstream signaling after integrin binding. Nevertheless, the molecular clutch model needs to be tested in 3D. Regarding mechanosensing by cell volume expansion, the general concept is that cells in 3D microenvironments respond to biophysical cues by increasing their volume and thus increasing the tension of their membrane, which causes the opening of ion channels, in particular TRPV-4 [82,83] or PIEZO1 [84,85] with subsequent intracellular ion influx. This ultimately leads to the activation of various biochemical metabolic pathways. However, the origin of the initial increase in cell volume remains to be clarified. Less information is available on the mechanotransduction of cells in relation to non-linear hydrogel mechanics. This aspect is of crucial importance since the cytoskeleton of cells can soften upon mechanical deformation, depending on the slippage of bonds or the breaking of cross-links within the cytoskeleton, as well as the buckling or severing of actin, but can also exhibit stiffening. Therefore, different biochemical cascades are activated/deactivated and the viscoelastic properties of the cells can be modulated depending on the loading conditions, both in terms of magnitude and time scales, type of application of stress/strain and loading history (mechanomemory) [28]. Regarding nonlinear elasticity, the work by Das et al. found a correlation between the onset of stiffening and the expression of the microtubule-associated protein DCAMKL1, implicating DCAMKL1 in a stress stiffening-mediated mechanotransduction pathway involving microtubule dynamics in stem cell osteogenesis [86]. In terms of plasticity, stress-relaxing hydrogels have been found to promote cell spreading, which is associated with local clustering of the substrate mediated by β1 integrin, actin polymerization, actomyosin contractility and increased YAP nuclear localization [19]. Regarding nonlinear viscoelasticity, hydrogels that exhibit strain-enhanced stress relaxation (SESR) have recently helped to elucidate for the first time the mechanotransduction processes in myoblasts. It was found that SESR activates integrin-focal adhesion kinase signaling and promotes F-actin polymerization. Ultimately, myogenic differentiation of myoblasts is enhanced by actin cytoskeleton polymerization-mediated myocardin related transcription factor (MRTF) nuclear localization and chromatin opening via nuclear mechanotransduction (e.g. nuclear morphology and histone acetylation) [87]. Nonlinear viscoelasticity was detected in normal skeletal muscle tissue, whereas it is almost absent in diseased skeletal muscle tissue (from Duchenne muscle dystrophy, DMD), suggesting that nonlinear viscoelasticity could also serve as a potential tool for screening physiological and non-pathological microenvironments. Further studies are needed to unveil the central role of both nonlinear elasticity, plasticity and nonlinear viscoelasticity in cell response in different tissues.

### 4.2 Review of non-linear hydrogel mechanics affecting cell behavior and activity

#### 4.2.1 Strain-softening

Strain-softening due to oscillatory shear has long not been a focus in cell mechanotransduction, as many ECM components such as collagen stiffen under mechanical stimulation [88], albeit strain-softening has also been identified [89]. However, the increased interest in dynamic hydrogels that can be plastically remodeled by cells following traction forces or more easily processed, e.g. as injectable or printable materials, combined with the evidence that living tissues such as liver can exhibit strain-softening under shear [[90], [91], [92]]. There are only a few examples of strain-softening hydrogels that have been explicitly developed for the mechanotransduction of cells in 2D and 3D culture conditions.

##### 4.2.1.1 Gelatin methacryloyl (GelMA) hydrogels

Bertsch et al. [39,45] have designed gelatin methacryloyl (GelMA) hydrogels with varying degrees of incorporated gelatin nanoparticles to modulate their stress relaxation. While pure GelMA hydrogels showed a linear elastic stress-strain response in a broad strain range, the incorporation of nanoparticles made hydrogels strain-softening at strains relevant for cells (10–50% in a three-dimensional microenvironment). This was associated with an accelerated non-linear viscoelastic stress relaxation at increasing strain (Fig. 2B and C) which promoted the spreading of pre-osteoblastic cells. Stress relaxation promotes the spreading of cells via integrin adhesions and actomyosin-based contractility which drive the nuclear translocation of transcriptional regulators YAP/TAZ [19,77]. The use of such particulate or colloidal hydrogels is a general trend for the design of hydrogels that facilitate cell invasion due to their more dynamic and typically strain-softening nature [[93], [94], [95], [96]]. Lipari and Marfoglia et al. [97] have exploited GelMA substrates for the investigation of cell adhesion. By photocrosslinking GelMA in a heated ("Hot", *T* = 37 °C) or cooled ("Cold", *T* = 4 °C) state, the authors developed a series of hydrogels with different mechanical properties, forming networks with an almost purely elastic (and soft) or viscoelastic (and rigid) behavior. Interestingly, Hot hydrogels show a linear stress/strain behavior up to a total deformation of about 10%, while Cold hydrogels enter the softening region at strains of less than 1%.

##### 4.2.1.2 Agarose hydrogels

Viscoplastic agarose hydrogels that exhibit strain-softening under oscillatory shear have been developed using two main approaches: (*i*) the addition of an uncrosslinked lactose-modified chitosan, forming semi-interpenetrating networks [98] or (*ii*) the use of agarose biopolymers with different chemical compositions in terms of the degree and pattern of methylation [13]. Both approaches allow the formation of hydrogel networks that exhibit fast stress relaxation in the order of tens of seconds, which is important for cellular activities, as well as low viscous energy dissipation (low loss tangents in the range of 4–6 %). When used as 2D substrates for cell adhesion, there is a threshold value for the elastic energy, $E_{e}$, expressed as J/m^3^ and calculated at the yield point, $\gamma_{c}$, as

eq. 16 $$E_{e}=\int_{\gamma =0}^{\gamma_{c}}\left({\frac{d\sigma }{d\gamma }|}_{\gamma =0}\right)\gamma d\gamma =\frac{1}{2}G\gamma_{c}^{2}$$

above which the adhesion to the substrate is hampered for different types of cells. Therefore, the elastic energy is a barrier to cell adhesion on viscoplastic substrates under 2D conditions. The elastic energy is a parameter that considers both an elastic component, namely the complex shear modulus, *G*, and a viscoelastic contribution that is $\gamma_{c}$. It is important to remember that the contribution of viscoelasticity outweighs the elasticity (the exponent is 2 for $\gamma_{c}$ and 1 for *G*). Therefore, soft hydrogels that show strain softening at very low critical strains would have very low $E_{e}$ values compared to rigid hydrogels that have higher critical strains. Taking all these considerations together, the lower $E_{e}$ is, the easier it is to enter the nonlinear stress-strain plastic region. Interestingly, the activities of the cells in terms of cell spreading, the formation of vinculin-rich focal adhesions and the mechanotransmission of traction forces correlated with the extent of strain-softening or plasticity, expressed as energy loss, $E_{l}$ (see eq. (8)), in the non-linear region [13]. Overall, the higher the plasticity of the substrate, the more cell functions are triggered. The effect of elastic energy and energy loss on cell activity, particularly with regard to cell spreading, invadopodia formation and migration, remains to be investigated in viscoplastic 3D agarose microenvironments.

##### 4.2.1.3 Alginate-based hydrogels

The concept of elastic energy has recently been applied to 3D confined microenvironments using an alginate methacrylate (ALMA) photo-crosslinked viscoelastic hydrogel network that exhibits strain-softening [53]. This gelling system allows deformations of up to 60% to be achieved under oscillatory shear within the linear stress-strain regime before entering the plastic region. A correlation between the amount of elastic energy needed to reach the critical strain and cellular stress conditions was demonstrated in the form of G3BP-mediated stress granule formation in an osteosarcoma cell line. This condition is associated with an increase in alkaline phosphatase (ALP) activity but a decrease in gene expression and is mediated by nuclear translocation of YAP. The elastic energy of the microenvironment thus exerts a mechanical feedback in 3D, which the cells perceive and respond to accordingly. A viscoplastic methylated alginate hydrogel network was developed using calcium sulfate as crosslinker and used as a platform for 3D cell studies. This hydrogel system exhibits fast stress relaxation, strain-softening under oscillatory shear with a critical strain of about 16% and permanent deformation (plasticity). Interestingly, this hydrogel platform promotes cell proliferation and nuclear translocation of YAP compared to a standard hydrogel made from a non-methylated alginate from *L. hyperborea* [52]. Lastly, nanoporous interpenetrating networks (IPNs) based on alginate and reconstituted basement membrane (BM) have been developed that exhibit an elastic-plastic transition with a yield stress <10 Pa, whereby the plasticity can be modulated independently of the stiffness [15]. In this set of hydrogels, cells in IPNs with high plasticity undergo protease-independent migration since they are able to expand their invadopodia to mechanically and plastically open microscopic channels and then migrate through these channels.

##### 4.2.1.4 Strain-stiffening

Strain- or stress-stiffening of biological networks can occur at short time scales immediately after leaving the linear stress-strain regime. It is important to recall that non-linear stiffening may not be fully reversible and is closely related to the fibrous architecture of the network. For a detailed description of non-linear stiffening of biological hydrogels (collagen and fibrin), we refer to a recent review on the subject [54]. There are different examples of non-linear viscoelastic hydrogels that stiffen over time in presence of cells [69,99]. These time-dependent non-linear viscoelasticity can be exploited to direct the differentiation of hMSCs towards osteogenic (early stiffening) or adipogenic (late stiffening), respectively [100]. In terms of stress-relaxation, Nam et al. [68] demonstrated that strain-stiffening collagen hydrogels show non-linear viscoelasticity, i.e., accelerated stress relaxation at increasing strain (Fig. 2G). Similar results were also reported by Andrée et al. [39] for collagen. Interestingly, several studies reported that collagen hydrogels might initially show strain-stiffening at intermediate shear strains, but ultimately strain-softening at higher shear strains, although typically beyond the strain range relevant for cells [39,88,101]. Nam et al. [16] also investigated the plasticity of several common cell culture materials, revealing that only strain-stiffening collagen and fibrin exhibit a stress-dependent non-linear plasticity. Contractile cells are able to remodel such collagen networks by the formation of new cross-links at small strains or by permanent fiber elongation at high strains [42]. A similar non-linear increase in plasticity at increasing creep stress was reported for polyisocyanide hydrogels [69]. Actually, the following synthetic hydrogel systems are described in literature to access the stresses/strains relevant for cell activities.

##### 4.2.1.5 Polyisocyanopeptide (PIC) hydrogels

Responsive biomimetic networks of polyisocyanopeptide (PIC) hydrogels with strain-stiffening properties similar to those of biological networks have been developed [55,102,103]. This set of hydrogels has recently been used as a platform to regulate cellular organization or decipher the mechanical interactions between cells and matrix [69]. The authors have shown that cellular contractile forces lead to remodeling and stiffening of the surrounding microenvironment via two different mechanisms. On short time scales, cellular contraction deforms the matrix, leading to an immediate stiffening response due to the non-linear stiffening properties of the matrix, which are directly linked to its fibrous architecture. It is worth noting that this effect is only temporary, and the stiffening of the matrix ceases when the load is removed. On longer time scales, the cells are able to remodel their matrix, compacting and contracting it, inducing plastic deformation. PIC-based hydrogels have also been used in combination with hyaluronic acid (HA-PIC) to give them antifibrotic properties [104]. Interestingly, the coupling of hyaluronic acid preserves non-linear stiffening. Biologically, proliferation and macroscopic contraction assays as well as stress fiber formation studies and characteristic fibrosis markers indicate a strong antifibrotic effect of the HA-PIC hydrogel. Stress-mediated stiffening has also been observed in similar hydrogel systems [86]. The results of this work show a correlation between the onset of stiffening and the commitment of adipose-derived stem cells (hASCs) that could be switched from adipogenesis to osteogenesis by changing the onset of stress stiffening.

##### 4.2.1.6 Bottlebrush hydrogels

Bottlebrush polymers, which consist of a linear polymer backbone from which the polymer side chains extend radially, can mimic the rheological properties of molecular filaments [105]. Among them, linear-bottlebrush-linear (LBL) triblock copolymers have been used to form plastomers and hydrogels that mimic the non-linear elastic bulk tissue mechanics. Recently, bottlebrush polymers were synthesized and crosslinked to form poly(ethylene glycol)-based hydrogels and used to study how strain-stiffening behavior affects human mesenchymal stromal cells (hMSCs) [106]. These hydrogels are not stress-relaxing, purely elastic, and not fibrous, which means that the strain-stiffening effects can be well decoupled from viscoelasticity. When cells are cultured in 3D in these hydrogels, they form focal adhesion-mediated protrusions due to actomyosin dynamics as a function of the critical stress that marks the onset of stress-stiffening.

##### 4.2.1.7 Lactose-modified chitosan hydrogels

Recently, a semi-synthetic lactose-modified chitosan has shown interesting macromolecular properties in the form of non-linear stiffening similar to natural biopolymers such as neurofilaments or collagen [107], and used as biomimetic extracellular matrix for the 3D encapsulation of cells [108]. Interestingly, lactose-modified chitosan was found to gel in the presence of boric acid, which behaves as a temporary crosslinker and promotes the formation of an out-of-equilibrium hydrogel through cycles of nucleation, reorganization, and disassembly. Remarkably, this hydrogel gelling system can be properly modulated by pH- or competitor-assisted mechanisms, but stiffening is preserved in the non-linear stress-strain regime [108,109]. However, mechanobiological studies are required to verify the effectiveness of the non-linear stiffening of this hydrogel network for the control of cell functions.

## 5 Conclusions and perspectives

Viscoelastic stress relaxation and plasticity have emerged as novel mechanical cues that have reshaped our understanding of cell mechanotransduction. This has triggered immense interest in viscoelastic hydrogels as ECM biomimetics for cell or organoid culture and tissue regeneration. However, the effect of hydrogel non-linearity on these viscoelastic properties has been largely neglected. In this perspective, we have compiled evidence from literature that hydrogel non-linearity can result in non-linear viscoelasticity, i.e., strain- or stress-dependent stress relaxation or plastic deformation. We found that both strain-softening and -stiffening result in accelerated stress relaxation as a function of strain, or increased degree of plasticity as a function of stress. Several common hydrogels described in literature are designed to be strain- or stress-sensitive in ranges relevant for cell activity, and thus also exhibit non-linear viscoelastic behavior in these regimes. Hence, the non-linearity of hydrogels in biologically relevant regimes is a potential new design parameter for hydrogels which can exhibit increased viscoelasticity in response to cell activity.

Rather than focusing on the standard application of an instantaneous stress/strain to enter the non-linear domain, we have focused here on the non-linear behavior of hydrogels accessible through oscillatory shear rheology and provided a simple mathematical modelling to identify it. There are two main reasons why oscillatory rheology could be very useful in the context of mechanotransduction of cells, especially when intended for 3D cell confinement studies: (*i*) cells continuously undergo polymerization/depolymerization cycles of their cytoskeleton, especially involving actin and microtubules; (*ii*) these cytoskeletal dynamics in turn remodel the surrounding microenvironment, leading to cumulative softening (plastic behavior) [14]. It is important to remember that irreversible remodeling of the microenvironment could be enhanced by the activity of metalloproteinases (MMPs) with concomitant degradation of extracellular collagen. Therefore, the analysis of hydrogels through an oscillatory mode would, in principle, resemble the cell dynamics in confined 3D microenvironments. A crucial question that needs to be examined in detail in the future is the extent to which macroscopic shear rheology is effectively related to cellular length scales and deformation modes. Importantly, the use of alternative micro- or nano-rheology tools, which work on very small scales, could provide key information to bridge this gap.

**Fig. 3:**
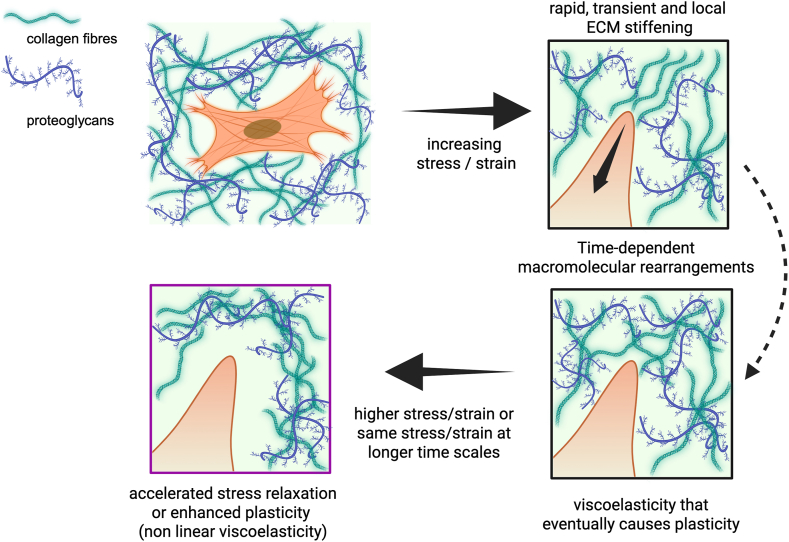
**Cartoon.** In living tissues, cells are embedded in a 3D microenvironment consisting of an intricate hydrogel network of structural proteins such as collagen and proteoglycans that form the extracellular matrix (ECM). Cells interact closely with the ECM and sense its biophysical properties while generating stress/strain cycles through polymerization/depolymerization of cytoskeletal components or volume expansion. At low stress, cells sense the immediate resistance of the ECM, i.e., linear elasticity or stiffness. The cells can produce an almost immediate, but transient, local non-linear stiffening due to the alignment of the ECM fibers. However, when the stress persists the ECM responds in a time-dependent manner (viscoelasticity). The ECM can eventually undergo plastic deformation when weak bonds in the ECM unbind, allowing the cells to remodel, or when ECM proteins molecularly slide, leading to an arrangement of the ECM by cell contraction. This results in a non-linear softening that causes a diffuse change in the ECM network. We propose that repeated cycles of polymerization/depolymerization of cytoskeletal components generating the same stress/strain on different time scales or gradually increasing their magnitude may lead to non-linear viscoelasticity effects, with enhanced stress relaxation and increased plasticity of the ECM. Created in BioRender. Sacco, P. (2025) https://BioRender.com/apwytrm.

Another important aspect of this perspective is the different stress/strain values relevant to cellular activities. While stiffening is known to occur at relatively low stress (<25 Pa) or strain values (<10%) [12,54,106], softening requires, often, larger strain ranges [15,[46], [47], [48], [49], [50]]. Therefore, cells would interact with the surrounding microenvironment, experience traction forces or volume expansion of different magnitudes and on different time scales, and perceive the corresponding mechanical feedbacks to direct their fate. When deforming the ECM in a single cycle of polymerization/depolymerization of the cytoskeleton, a cell would thus experience the following mechanical feedbacks as a function of time: (*i*) an almost instantaneous and fully reversible elastic response due to its resistance to deformation; (*ii*) a transient, local and mostly reversible, non-linear stiffening on short time scales; (*iii*) a time-dependent macromolecular rearrangement causing creep and stress relaxation that eventually leads to permanent non-linear softening (plastic remodeling), a scenario consistent with what has been recently proposed [9]. (*iv*) Here we emphasize that further polymerization/depolymerization cycles applying the same stress/strain on longer time scales or progressively increasing the stress/strain can enhance stress relaxation or increase plasticity, leading to non-linear viscoelasticity (Fig. 3, Cartoon). From a biomaterial perspective, this represents an interesting challenge for future studies in this field. The main objective would therefore be to recapitulate the non-linear responses on different time scales in a unique dynamic hydrogel network, with particular emphasis on non-linear viscoelastic effects, and to investigate their impact on cellular functions.

## CRediT authorship contribution statement

**Pascal Bertsch:** Writing – original draft, Visualization, Investigation, Conceptualization. **Pasquale Sacco:** Writing – original draft, Visualization, Investigation, Conceptualization.

## Notes

The authors declare no conflict of interest.

## Declaration of competing interest

The authors declare no conflict of interest.

## Data Availability

All data reproduced in this review is based on previously published data as referenced
